# Exploring the impact of landscape environments on tourists’ emotional fluctuations in Fujian’s Coastal National Parks using machine learning

**DOI:** 10.1371/journal.pone.0329118

**Published:** 2025-08-13

**Authors:** Zekun Lu, Shunhe Chen, Chao Qiu, Rongxiang Chen, Yuchen Lin, Yichen Lu, Ying Xu

**Affiliations:** College of Arts College of Landscape Architecture, Fujian Agriculture and Forestry University, Fuzhou, Fujian Province, China; Zhejiang Agriculture and Forestry University: Zhejiang A and F University, CHINA

## Abstract

In recent years, the impact of landscape environments on tourists’ emotions has increasingly become a significant topic in sustainable tourism and urban planning research. However, studies on the relationship between multidimensional environmental features of Coastal National Parks and tourists’ emotions remain relatively limited. This study integrates machine learning and multi-source data to systematically explore how the landscape environments of Fujian’s Coastal National Parks influence tourists’ emotional fluctuations. Using natural language processing (NLP) techniques, sentiment indices were calculated from social media textual data, while semantic segmentation models and image analysis were employed to extract environmental feature data. The Light Gradient Boosting Machine (LightGBM) model and SHapley Additive exPlanations (SHAP) method were used to evaluate the relative importance of different environmental variables on tourists’ emotions, with the findings visualized using ArcMap. The results indicate: (1) Over the past five years, 87.06% of emotions were positive, with the highest sentiment indices observed in the Fuyao Islands, Changle, and Xiamen. (2) Greenness (0.0–0.2) and aquatic rate (0.1–0.15) had the most significant positive impacts on emotions, whereas transportation proportion and paving degree had relatively minor effects. This study provides a theoretical basis for the sustainable development of Coastal National Parks and offers practical insights for optimizing landscape planning to enhance tourists’ emotional experiences.

## 1. Introduction

In recent years, marine protected areas (MPAs) have been widely adopted worldwide as critical public policy instruments to address coastal environmental pressures, halt the loss of marine biodiversity, and manage the impacts of human activities [1]. Among these, marine parks that combine ecological protection with public service functions have been gradually established in coastal regions of various countries. Port-Cros National Park in France, one of the earliest marine national parks to be established in Europe, highlights how the evolution of governance models has underscored the importance of local community acceptance and participatory mechanisms in achieving conservation effectiveness [2]. Cape Lookout National Seashore in the United States utilized spatial social value modeling tools to examine the spatial preferences of various user groups for ecological and cultural services, providing empirical insights for multi-objective coastal management [3]. Research on Machalilla National Park in Ecuador suggests that the differentiation of ecotourism motivations and the segmentation of visitor demands play a constructive role in balancing ecological protection and community development [4]. In China, according to the State Oceanic Administration, Coastal National Parks—classified as a type of marine special protected area—emphasize strict conservation of marine ecological and cultural resources. These parks are demarcated by the state in accordance with the law and are designed to integrate public functions such as scientific research, environmental education, and ecological recreation. The 19th National Congress of the Communist Party of China proposed twice the establishment of a protected natural area system centered on national parks [5]. The revised “Marine Special Protected Area Management Measures” by the State Oceanic Administration officially incorporated Coastal National Parks into marine special protected areas [6]. Within this policy context, the development of Coastal National Parks is regarded as a crucial approach to reconciling socio-economic development needs with the goal of maximizing the integrity and stability of regional ecosystems. It provides a systematic institutional framework for ecological conservation and resource management in coastal areas [7].

Recently, ecotourism, as a key form of sustainable tourism, has been recognized as one of the fastest-growing sectors in the global tourism industry [8]. It has an average annual growth rate of approximately 5%, which is nearly three times that of conventional tourism [9]. Coastal National Parks play a crucial role in ecological conservation and biodiversity restoration. They have also become key drivers of regional economic development [10],particularly serving as vital pillars of the ecotourism industry [4]. The landscape environment of Coastal National Parks, as a core factor in the tourist experience, significantly influences tourists’ emotional responses and behavioral patterns [11]. In international research on the management and tourism of Coastal National Parks, scholars have approached the topic from multiple dimensions. García‐Melón et al. employed the Analytic Network Process (ANP) method to assess sustainable tourism strategies for Venezuela’s Coastal National Parks and proposed optimized management pathways grounded in systematic decision-making [12]. Carvache-Franco et al. conducted fieldwork and surveys to analyze ecotourism motivations and market segmentation among visitors to Coastal National Parks, providing empirical evidence for improving tourism products and promoting sustainable destination development [4]. Chapungu et al. integrated temperature and fire monitoring data with triangulation and convergence modeling to examine climate change trends and their potential impacts on tourism activities across six Coastal National Parks in South Africa, revealing the challenges posed by climate factors to the stability of tourism systems [13]. Additionally, Sung-Hee et al. utilized social media data to extract visitor perceptions of specific landscapes in Korea’s Taean Coast National Park. Although their analysis revealed preferences among visitors, it did not incorporate computer vision techniques such as semantic segmentation for the quantitative identification of landscape elements [14]. Although existing research has produced substantial findings on tourist motivation, management, and social perceptions, there remains a lack of systematic theoretical frameworks and empirical studies on how environmental factors influence tourist emotions and behaviors through visual and linguistic cues—particularly from a multimodal perspective that integrates both images and text. This area calls for more in-depth investigation.

Emotion refers to a complex physiological and psychological response triggered by specific external stimuli [15]. The global prevalence of mental health issues was highlighted by the World Health Organization in 2013. As a direct reflection of mental states, emotion has become a major research focus in fields such as psychology and cognitive neuroscience [16]. With the development of society and economy and the intensification of urbanization, people’s values have shifted from material to spiritual needs, sparking interest in returning to nature and appreciating the natural landscapes of Coastal National Parks [17]. Exposure to natural marine landscapes significantly alleviates stress and enhances health [18]. Coastal National Parks leverage the unique geographical and aesthetic value of marine natural landscapes to promote the sustainable use of ecological resources and economic development [19]. Therefore, the Chinese government has implemented a series of policies to enhance marine tourism development, such as improving the allocation of marine resources, advancing high-quality tourism, and protecting the marine environment [20]. Additionally, tourists’ overall emotional experiences during travel are influenced by environmental factors [21], and the sustainable economic development of tourist destinations largely depends on positive tourist experiences [22]. Emotional Geography, a subfield of geography, explores the relationships between emotions, space, place, and environment. It reveals that environmental quality significantly affects emotional well-being, with high-quality environments fostering positive emotions [23]. Different environments significantly influence human emotions, thereby altering the overall experience of activities [24]. Studies on the relationship between the built environment and public mental health indicate that the quality of spatial environments significantly impacts public emotions and mental health symptoms [25]. Therefore, considering the impact of the environment on individual emotions is crucial in planning and design. Studying personal perception preferences of Coastal National Park landscapes aligns with the United Nations’ Sustainable Development Goals (SDGs) for conserving marine resources, communities, health, and well-being [26].

The evaluation of spatial elements is a critical component in studies of spatial emotion and visitor satisfaction [27]. Current tools such as the Public Space Public Life (PSPL) method [28], the ParkScore Index [29], and the Post-Occupancy Evaluation (POE) framework [30] are widely used in practice. However, they still face limitations in integrating multimodal data and analyzing complex perceptual responses [31]. With the advancement of big data and machine learning, research on urban spatial quality has increasingly shifted toward multisensory dimensions, including visual perception [32]. The rise of social media platforms has made user-generated content (UGC) a valuable data source for analyzing tourist behavior, preferences, and emotional states [33]. UGC includes diverse formats such as text, images, videos, reviews, and ratings, and is widely distributed across platforms like Weibo, Mafengwo, Ctrip, and Dianping [34]. It has been extensively used in research on landscape sustainability, human-environment relationships, and ecosystem monitoring [35]. UGC provides a large volume of effective data for understanding tourist travel motivations, environmental perceptions, and emotional experiences [36]. Studies based on UGC have revealed how multiple factors—including marine features, accessibility, management practices, and spending levels—jointly influence tourist preferences and experiences in Coastal National Parks [35]. Typical examples include: Zhu et al. [37]’s study on visitor behavioral intentions in marine theme parks; Hurtado et al. [38]’s research on satisfaction and crowding perception in the Florida Keys National Marine Sanctuary; and Bidegain et al. [39]’s visual analysis of cultural landscape preferences and ecosystem service perceptions in the Mediterranean region. Moreover, UGC has demonstrated significant advantages in studies related to tourism planning, landscape perception, the emotional impact of environmental quality, and destination vitality [40,41]. These studies have further expanded the perspectives and methodological frameworks of tourism landscape research. However, most existing UGC-based research remains focused on large-scale urban or natural environments [42,43], while multimodal and integrative studies on small- to medium-scale natural environments—particularly Coastal National Parks—are still relatively scarce. Against the backdrop of rapid tourism development and mounting ecological pressures on coastal parks, these gaps highlight the urgent need for more practical and robust data processing frameworks in related research domains.

As the demand for multi-source data integration and analysis grows in spatial environmental research, machine learning methods have been widely adopted to address the challenges posed by data complexity [44]. Among these, the Gradient Boosting Decision Tree (GBDT) has become one of the most commonly used modeling tools in spatial ecological research due to its flexibility and high efficiency [45]. GBDT constructs multiple weak learners iteratively, gradually minimizing prediction errors and thereby improving the overall model fitting accuracy [46]. This feature makes it particularly suitable for handling high-dimensional, nonlinear, and multivariate environmental data, demonstrating strong adaptability in fields such as environmental feature quantification and spatial-emotion relationship modeling [47,48]. The LightGBM framework, built on GBDT, further enhances computational efficiency and accuracy, and supports large-scale parallel training [49]. Currently, LightGBM has been widely applied in regional environmental studies, including urban development strategies [50], land cover classification [51], and scenic area safety [52]. In addition, LightGBM can extract high-dimensional features from UGC, identify key variables affecting tourists’ emotional responses, and offer interpretable results through feature importance ranking [53]. This capability enables researchers to identify which landscape features have the greatest influence on emotional responses and to quantify the contribution of each variable to emotional indices [54]. Although this method has been widely used in urban planning and landscape studies [55], it still falls short in identifying and explaining variables strongly associated with emotional experiences [56]. Spatially representative information such as regional openness, image complexity, and remote sensing data still lacks refined indicators and contextual environmental interpretation, limiting its applicability in complex Coastal National Park settings. Due to the inherent “black-box” nature of traditional machine learning models, which limits the ability to accurately identify the contribution of individual variables during modeling, researchers have increasingly combined LightGBM with SHAP to enhance model interpretability and decision support [57]. Therefore, this study integrates textual and visual image data to better understand the relationship between emotional responses and macro-level spatial data. By incorporating multimodal data, this study aims to address the limitations of previous research in linking emotional responses with spatial data, offering a novel approach to exploring the relationship between tourists’ emotional experiences and macro-environmental features in Coastal National Parks.

In summary, the relationship between tourists’ emotional responses and spatial environments in Coastal National Parks remains underexplored, particularly regarding the mechanisms linking built environment features and emotional feedback from a multimodal data perspective. Therefore, this study focuses on representative coastal national marine parks in Fujian Province and proposes the following research objectives: First, to construct a multimodal data analysis framework by integrating user-generated images and textual content, extracting tourists’ emotional responses to landscape environments, and developing a perceptual system for analyzing emotion–space relationships. Second, to identify key elements of the built environment by using the LightGBM model to quantify spatial variables affecting tourist emotions, and applying the SHAP method to interpret each variable’s relative contribution. Finally, to examine the mechanisms between spatial environments and emotional responses by analyzing how different types of built environments in coastal parks shape tourists’ emotional fluctuations, thereby offering theoretical and practical insights for emotion-oriented spatial planning and management. To achieve these goals, this study combines social media data with machine learning methods to explore the relationship between tourists’ emotional responses and environmental variables in marine parks. The specific research steps are as follows:

1. Collect and clean image and textual data from platforms such as Ctrip, Mafengwo, and Dianping;
2. Perform sentiment analysis using Bidirectional Encoder Representations from Transformers (BERT) and Robustly Optimized BERT Approach (RoBERTa) architectures based on Baidu’s ERNIE multimodal pretrained model, and calculate the average sentiment index;
3. Image complexity was calculated in MATLAB; then the Pyramid Scene Parsing Network (PSPNet) was applied for semantic segmentation, and ArcMap was used to visualize environmental indicators for each park;
4. Construct the LightGBM model.
5. Use the SHAP method to interpret the influence mechanisms of environmental variables on tourists’ sentiment scores.

The remaining sections are organized as follows. Section 2 presents the theoretical framework, study area, and data sources, detailing the collection and preprocessing of textual and image data, as well as the multimodal modeling approach. Section 3 presents the empirical results, focusing on the contribution of various built environment variables to tourists’ sentiment scores and their underlying influence mechanisms. Section 4 proposes spatial optimization strategies based on emotional insights and discusses the theoretical implications, practical value, and limitations of the study. Section 5 concludes the study by summarizing key findings and outlining directions for future research.

## 2. Data and methodology

### 2.1. Study area

**Fig 1:**
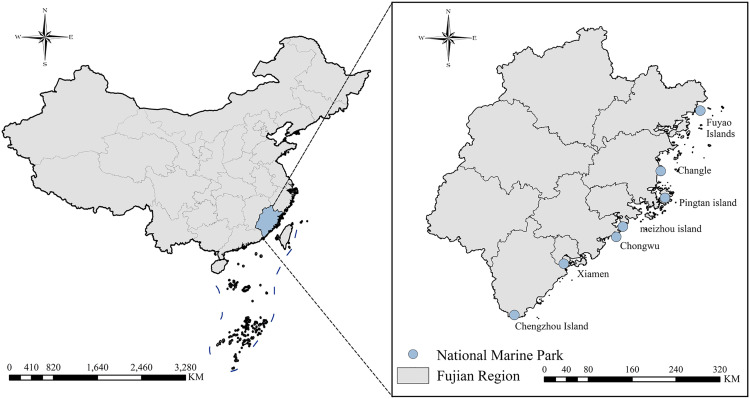
Study area.

Fujian Province is located along the southeastern coast of China and features a subtropical monsoon climate. It is a representative coastal province with a land area of 124,000 square kilometers, a sea area of 136,000 square kilometers, and a mainland coastline of 3,752 kilometers, accounting for approximately 16% of the national total, ranking second in China. As shown in Fig 1, the scope of this study includes seven national-level Coastal National Parks in Fujian Province. Thanks to its unique geographical and climatic advantages, Fujian Province has established seven Coastal National Parks and boasts the highest vegetation coverage rate in China [58]. In recent years, excessive urban expansion and intensive development have caused damage to the marine natural environment, creating conflicts between the development of Coastal National Parks and environmental conservation [59]. Additionally, the lack of specialized management personnel in Coastal National Parks has raised concerns about potential ecological degradation. In conclusion, this study focuses on the Coastal National Parks in Fujian Province, analyzing tourists’ emotional responses to landscape environments and actively proposing recommendations for improving the utilization and development of tourism resources in Coastal National Parks.

### 2.2. Theoretical framework

**Fig 2:**
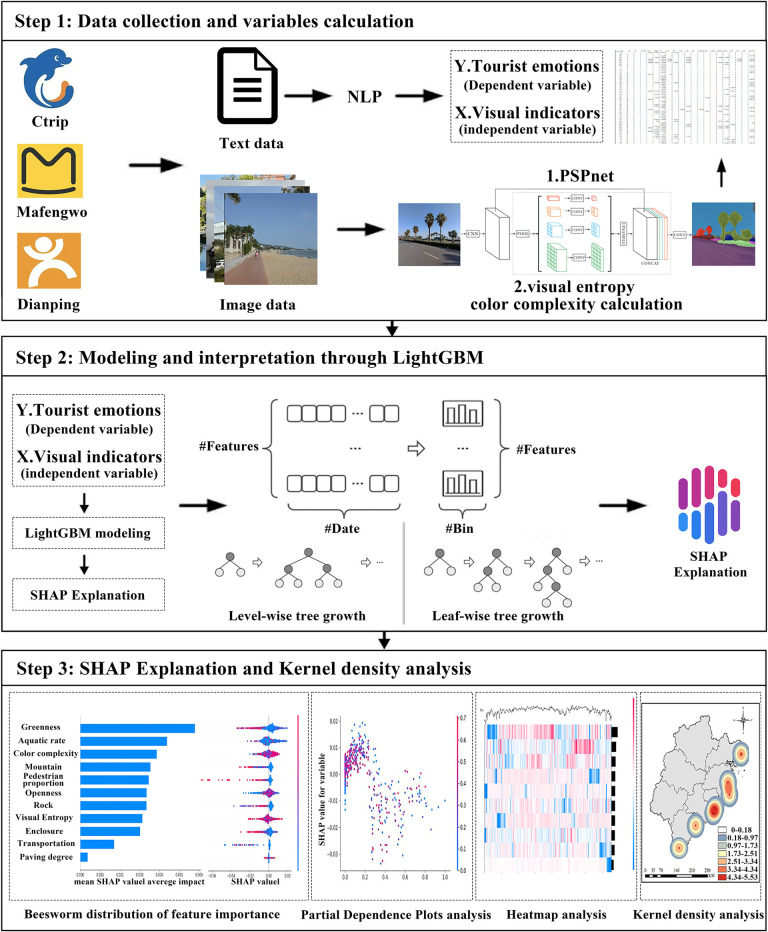
Theoretical framework.

This study is grounded in the theoretical frameworks of Environmental Psychology and Emotional Geography, aiming to explore the relationship between built environment characteristics of Coastal National Parks and tourists’ emotions, as well as the specific contributions of various environmental factors to overall emotions. The fundamental theory of Environmental Psychology posits that the built environment can influence individuals’ emotions and behavioral responses through multimodal perceptions, such as visual and emotional cues [60,61]. Meanwhile, the theoretical framework of Emotional Geography emphasizes the complex interactions between emotions, space, and place, exploring how specific environments evoke human emotional responses, which in turn influence the construction of spatial meaning and behavioral patterns [62]. Based on this, the study designed a multi-stage evaluation model combining UGC and machine learning, as illustrated in Fig 2, to reveal how the built environment of Coastal National Parks affects tourists’ emotional experiences. First, text and image data generated by users on tourism social media platforms such as Ctrip, mafengwo.com, and Dianping were collected. Second, the extracted image data were cleaned and categorized, and relevant visual elements of Coastal National Parks were identified using a semantic segmentation model based on the PSPNet. Additionally, Matlab tools were used to analyze the color and visual complexity of the image data. Third, text data extracted from tourism social media platforms were analyzed using NLP models based on BERT and RoBERTa, both trained on Baidu’s ERNIE multimodal pretraining technology. Sentiment indices were then scored. Finally, a LightGBM model was constructed, with the extracted independent and dependent variables incorporated into the model. The SHAP method was then used to interpret the model results. The study results were visualized and analyzed using ArcMap software.

### 2.3. Data collection

The data used in this study on the Coastal National Parks in Fujian Province were derived from the directory of seven national-level Coastal National Parks published by the State Oceanic Administration of China and other relevant government agencies. The parks included Chengzhou Island, Xiamen, Chongwu, Meizhou Island, Pingtan Island, Changle, and Fuyao Islands. To analyze tourists’ emotional fluctuations and experiences, the research team further collected relevant data from major Chinese tourism social media platforms. Social media data were gathered through keyword searches conducted between August 2019 and August 2024, covering text and image data related to the seven Coastal National Parks mentioned above. Data collection was conducted using Python-based web scraping techniques, primarily from the following three platforms: (1) Mafengwo (www.Mafengwo.com): A social platform primarily targeting travelers, where users share travel journals, attraction photos, and itinerary plans. The data collected mainly includes text and images. The platform has over 100 million users and is particularly popular among young travellers. (2) Dianping (www.dianping.com): A comprehensive local service platform covering dining, hotels, and tourism. Users share personal experiences through reviews and ratings. As of 2024, the platform has 18.9 million daily active users, widely covering major cities across China. (3) Ctrip (www.ctrip.com): The largest online travel platform in China, offering services such as flights, hotels, and travel packages. Users share reviews of travel products and services, including text and images. With over 400 million users, it is a key player in China’s tourism market. Using web scraping techniques on these platforms, the research team initially collected 3,914 data records containing both images and comments, reflecting tourists’ evaluations and feedback on the seven Coastal National Parks in Fujian Province. During the data cleaning phase, 156 invalid or low-quality entries—such as duplicates, empty comments, or irrelevant content—were removed. The remaining 3,758 valid records were retained for analysis. The cleaning process included deduplication, formatting, outlier handling, and noise removal to ensure consistency and accuracy across text, images, and metadata. For the image data, we also made efforts to maintain consistency in camera angles wherever possible to support reliable visual analysis.

### 2.4. Data compliance statement

The UGC used in this study, including images and textual reviews, was obtained from publicly accessible social media platforms such as Ctrip, Mafengwo, and Dianping. Only publicly available information was extracted during data collection, with no access to private user data, account information, or sensitive content. All data collection and analysis procedures strictly adhered to the data usage policies and terms of service of the respective platforms. The data were used exclusively for academic, non-commercial purposes. Furthermore, the research team did not engage in any manual intervention or content manipulation. All data were anonymized and analyzed statistically within an academic framework.

### 2.5. Selection of variables

This study builds upon previous research on the impact of the built environment on human emotions [63–65] to investigate how the built environment in Coastal National Parks influences tourists’ emotions. The selected indicators in this study were determined by considering the availability of key data as well as the relationship between the built environment and landscape features. Definitions and quantification methods for these indicators are detailed in Table 1. Among these indicators, traffic flow, enclosure, pedestrian proportion, and paving degree are categorized as anthropogenic indicators [66], while openness, aquatic rate, greenness, rock, and mountain are classified as natural indicators [67–69]. In studies of emotional perception, color attributes are considered key determinants of the visual continuity or contrast between landscape elements and their surrounding environment, making them critical factors influencing aesthetic evaluation and emotional responses [70]. Visual entropy, as an indicator of image information complexity, reflects the diversity and uncertainty of visual elements in the environment. It indirectly influences individuals’ cognitive load and emotional states [71]. However, previous studies have rarely addressed visual quality variables in the built environment in a systematic manner [32], this study specifically incorporates visual factors such as color complexity and entropy to evaluate their role in stimulating emotions.

**Table 1:** Research Element Breakdown Table.

Research Elements	Research indicators	Indicator Description	Quantitative methods
**Built environment**	Visual Entropy	Entropy value of images	Matlab
	Color complexity	Color entropy value of images	Matlab
	Mountain	The proportion of mountain in the image	PSPNet
	Aquatic rate	The proportion of aquatic rate in the image	PSPNet
	Pedestrian proportion	The proportion of pedestrians in the image	PSPNet
	Transportation	Transportation	PSPNet
	Paving degree	The proportion of pavement in the image	PSPNet
	Greenness	The proportion of green plants in the image	PSPNet
	Openness	The proportion of sky in the image	PSPNet
	Enclosure	Environmental building enclosure degree	PSPNet
	Rock	The proportion of rock in the image	PSPNet
**Tourist emotions**	Sentiment index	Text sentiment prediction	NLP

The analysis conducted using Matlab software yielded visual color complexity and its entropy values. By utilizing a PSPNet -based semantic segmentation model, image data were processed to quantify multidimensional built environment data. These data include indicators such as traffic flow, pedestrian proportion, aquatic plant coverage, ground paving degree, mountain, rock, greenness, openness, and enclosure. Additionally, sentiment indices were calculated by applying NLP models to text data. Each image data entry was paired with its corresponding text data to ensure the effective integration of multimodal data.

### 2.6. Data processing

#### 2.6.1. Sentiment analysis using pre-trained NLP models.

This study employs NLP models trained using Baidu’s ERNIE multimodal pretraining technology, including the BERT and the RoBERTa architectures. These techniques have outperformed the current state-of-the-art (SOTA) in 14 benchmark tasks for bilingual sentiment analysis in Chinese and English, with results published in ACL 2020 [72]. In addition, Baidu’s research team proposed the Sentiment Knowledge Enhanced Pre-training for Sentiment Analysis (SKEP) algorithm, a sentiment knowledge-enhanced pretraining method. The algorithm automatically mines sentiment knowledge and incorporates it into pretraining objectives, enabling the model to achieve a deeper understanding of sentiment semantics and further improve the performance of sentiment analysis tasks [73]. This study collected text and image data related to seven national-level Coastal National Parks from three tourism social media platforms: Mafengwo, Dianping, and Ctrip. After data cleaning, 3,758 valid entries containing image data were obtained. Finally, the text data were input into the NLP models for computation and analysis, with the results scored on a scale of 1–10, where higher scores indicate better emotional experiences. Additionally, the average sentiment score was calculated for each national-level Coastal National Park.

#### 2.6.2. Semantic segmentation based on the PSPNet model.

PSPNet incorporates a Pyramid Pooling module that extracts both global and local contextual information at multiple scales, enabling better understanding of semantic content in images and improving segmentation performance [74].‌Semantic segmentation is a technique in computer vision that assigns a semantic label to each pixel in an image, indicating the category it belongs to [75]. This study employed the PSPNet model with a ResNet-101 backbone pretrained on ImageNet and fine-tuned on the ADE20K dataset using 60 epochs of SGD training (momentum = 0.9, weight decay = 0.0001, initial learning rate = 0.001 with polynomial decay). Data augmentation included random scaling, flipping, and cropping, which helped the model adapt to the complexity of natural landscape scenes. Image data related to seven national-level coastal parks were collected from tourism-focused social media platforms, and the model was applied to extract quantitative values of various built environment features. In this study, the PSPNet model was applied to image data collected from seven national-level Coastal National Parks on tourism social media platforms to calculate values for various influencing factors of the built environment. In addition, mathematical formulas from previous studies were utilized to calculate factors such as sky openness, greenness, and aquatic rate [76,77]. Detailed quantitative explanations are provided in Table 2. In the final experiment, the PSPNet model achieved a mean Intersection over Union (mIoU) of 43.3% and a pixel accuracy of 80.8%. While these results may seem modest in absolute terms, they are in line with benchmark performances on the ADE20K dataset reported in previous studies (e.g., FCN: 41.6%, DeepLab: 44.1%, PSPNet: 44.94%) [74,78]. Given the complexity of the user-generated images used in this study—characterized by varied perspectives, lighting conditions, and mixed natural and built environments—this level of segmentation accuracy is considered acceptable for the purpose of environmental feature extraction.

**Table 2:** Semantic segmentation recognition explanation.

Research indicators	Explanation
**Mountain (M)**	Formula: M = (P_Mountain/ P_Total) × 100% Description: M represents the percentage of mountain pixels in the image, where P_Mountain is the pixel count of mountain elements and P_Total is the total pixel count in the image. Example: For an image with 500 mountain pixels out of a total of 5000 pixels, M = (500/ 5000) × 100% = 10%.
**Aquatic rate (A)**	Formula: A = (P_Aquatic/ P_Total) × 100% Description: A represents the percentage of aquatic pixels in the image, where P_Aquatic is the pixel count of aquatic elements. Example: For an image with 800 aquatic pixels out of 10,000 pixels, A = (800/ 10,000) × 100% = 8%.
**Pedestrian proportion (P)**	Formula: P = (P_Pedestrian/ P_Total) × 100% Description: P represents the percentage of pedestrian pixels in the image, where P_Pedestrian is the pixel count of pedestrian elements. Example: For an image with 2000 pedestrian pixels out of 15,000 total pixels, P = (2000/ 15,000) × 100% = 13.33%.
**Transportation (T)**	Formula: T = (P_Transportation/ P_Total) × 100% Description: T represents the percentage of transportation pixels in the image, where P_Transportation is the pixel count of transportation elements. Example: For an image with 1200 transportation pixels out of 10,000 pixels, T = (1200/ 10,000) × 100% = 12%.
**Paving degree (PA)**	Formula: PA = (P_Paving/ P_Total) × 100% Description: PA represents the percentage of paving degree pixels in the image, where P_Paving is the pixel count of paving degree elements. Example: For an image with 3000 paving pixels out of 15,000 pixels, PA = (3000/ 15,000) × 100% = 20%.
**Greenness (G)**	Formula: G = (P_Greenness/ P_Total) × 100% Description: G represents the percentage of greenness pixels in the image, where P_Greenness is the pixel count of greenness elements. Example: For an image with 1200 greenness pixels out of 20,000 pixels, G = (1200/ 20,000) × 100% = 6%.
**Openness (O)**	Formula: O = (P_Openness/ P_Total) × 100% Description: O represents the percentage of openness pixels in the image, where P_Openness is the pixel count of openness elements. Example: For an image with 1800 openness pixels out of 18,000 pixels, O = (1800/ 18,000) × 100% = 10%.
**Enclosure (E)**	Formula: E = (P_Enclosure/ P_Total) × 100% Description: E represents the percentage of enclosure pixels in the image, where P_Enclosure is the pixel count of enclosure elements. Example: For an image with 1000 enclosure pixels out of 15,000 pixels, E = (1000/ 15,000) × 100% = 6.67%.
**Rock (R)**	Formula: R = (P_Rock/ P_Total) × 100% Description: R represents the percentage of rock pixels in the image, where P_Rock is the pixel count of rock elements. Example: For an image with 500 rock pixels out of 20,000 pixels, R = (500/ 20,000) × 100% = 2.5%.

#### 2.6.3. Color complexity and visual entropy calculation using MATLAB.

Visual entropy is a statistical measure used to quantify the amount of information in an image, reflecting the richness of the information it contains. In image processing, visual entropy can be calculated using MATLAB tools for grayscale enhancement and segmentation, by estimating the probability of occurrence for each grayscale value in the image. In the processed images, Formula 1 for visual entropy defines n significant boundary units or regions. i represents the segmented regions, while $P(a_{i})$ denotes the probability of occurrence for region $a_{i}\;(i=1,2,\ldots ,n)$. Additionally, H(x) represents the total amount of information produced by a complete visual object composed of n regions. Visual entropy is defined based on the Shannon entropy method. Shannon entropy is essentially a method for measuring the uncertainty and information content of a system, widely applied in fields such as information science and ecology. In this study, the application of visual entropy focuses on image processing, aiming to quantify the informational complexity and distribution characteristics of visual objects [71,79]. Although its calculation method is similar to that of Shannon entropy, the concept of “visual entropy” is specifically tailored for analyzing grayscale distributions in images to reflect the richness of the information they contain. In addition, the study also employs the color complexity calculation, referred to as Formula 2, to evaluate the spatial distribution complexity of different colors in the images. The definition of color complexity is as follows: where N represents the total number of pixels for a specific color; m denotes the number of connected regions in the general set; and $C_{k}$ represents the complexity of spatial distribution for a specific color; $n_{i}$ represents the number of pixels in the i connected region, and this formula is used to describe the information content of color spatial distribution. This formula is used to describe the information content of the spatial distribution of colors. When the color distribution is more uniform and regions are more dispersed, the color complexity value $C_{k}$ is higher [80]. The joint analysis of visual entropy and color complexity enables a more comprehensive characterization of the statistical properties of grayscale and color distributions in images.


$$\begin{array}{c} H(x)=-\sum {\mathrm{\backslash nolimits}}_{i=1}^{n}P(a_{i})*logP(a_{i}) \end{array}$$ (1)

$$\begin{array}{c} C_{k}=-\sum {\mathrm{\backslash nolimits}}_{i=1}^{m}n_{i}\log\left(\frac{n_{i}}{N}\right) \end{array}$$ (2)

#### 2.6.4. Kernel density analysis based on Arcmap.

This study utilized the Geographic Information System (GIS) tool ArcMap 10.8.1 to analyze and visualize spatial data from Fujian’s Coastal National Parks, aiming to reveal the spatial characteristics of tourist sentiment predictions and landscape preference distributions. ArcMap, developed by the Environmental Systems Research Institute (ESRI), is a GIS platform widely used for spatial analysis, cartography, and spatial database management. In this study, ArcMap 10.8.1 was used to perform kernel density analysis and visualize geographic data, providing essential technical support for analyzing landscape preferences in the study area. Kernel density analysis is a statistical tool used to measure and analyze the density of point or line features within their surrounding neighborhoods, interpreting the spatial relationships of data density within a region. The concept of density analysis is based on overlaying a smooth surface over each feature, where the surface value is highest at the feature’s location and decreases gradually with increasing distance. At a distance equal to the search radius, the surface value becomes zero. In this study, the quantified research elements were assigned to point coordinates, and kernel density calculations were conducted to visually display the distribution of landscape preferences across seven Coastal National Parks in Fujian. Formula 3 in this study defines i as a data point (i=1,…,n) and assigns corresponding built environment indicator data to each data point. The parameter ${\text{pop}}_{i}$ is optionally used to describe the assignment of data point i, while ${\text{dist}}_{i}$ represents the distance between the data point and the coordinate location (x,y) [81]. Through kernel density analysis, this study reveals the spatial distribution characteristics of landscape preferences across seven Coastal National Parks in Fujian and explores the relationship between tourist emotions and landscape features. Each data point was assigned corresponding built environment indicators to further analyze the spatial correlation and distribution patterns of variables.


$$\begin{array}{c} \text{Density}=\frac{1}{{\left(\text{radius}\right)}^{2}}\sum {\mathrm{\backslash nolimits}}_{i=1}^{n}\left[\frac{3}{\pi }\cdot {\text{pop}}_{i}{\left(1-{\left(\frac{{\text{dist}}_{i}}{\text{radius}}\right)}^{2}\right)}^{2}\right] \end{array}$$ (3)

#### 2.6.5. LightGBM model construction and SHAP analysis.

This study employs the LightGBM algorithm to construct a regression model and explore the correlation between various research indicators in Coastal National Parks and human emotions. Compared to traditional Gradient Boosting Decision Tree (GBDT) models, LightGBM offers highly efficient parallel training, faster training speeds, lower memory usage, better accuracy, and support for distributed computing, enabling the rapid processing of large datasets [82]. Equation 4 demonstrates the calculation process for the split gain in LightGBM. $G_{L}$ and $G_{R}$ represent the gradient sums of the left and right subtrees, $H_{L}$ and $H_{R}$ represent the second-order derivative sums of the left and right subtrees, λ is the regularization parameter used to prevent overfitting, and γ is the penalty term for the number of leaf nodes. However, such models are often considered black boxes, as their construction process and the contribution of different variables are not directly visible. The SHAP tool offers precise explanations by quantifying each variable’s contribution to the model. SHAP is a method for interpreting individual predictions by calculating the contribution of each feature to the predicted variable. It computes Shapley values based on cooperative game theory. Therefore, this study evaluates the contribution of each indicator by calculating the contribution values of independent variables (natural landscape features) to the dependent variable (sentiment index). In Equation 5, g(z′) represents the predicted sentiment index influenced by the landscape feature z′, ∅0 denotes the average indicator composed of landscape features, M represents the number of variables in the model, and ∅i represents the SHAP value of the i research indicator.


$$Gain=\frac{1}{2}\left[\frac{{\left(G_{L}+G_{R}\right)}^{2}}{H_{L}+H_{R}+\lambda }-\frac{G_{L}^{2}}{H_{L}+\lambda }-\frac{G_{R}^{2}}{H_{R}+\lambda }\right]-\gamma$$ (4)

$$\begin{array}{c} g(z^{\prime })=\emptyset_{0}+\sum {\mathrm{\backslash nolimits}}_{i=1}^{M}\emptyset_{i}{z^{\prime }}_{i} \end{array}$$ (5)

## 3. Results

### 3.1. Distribution of tourist sentiment indices across different regions

**Fig 3:**
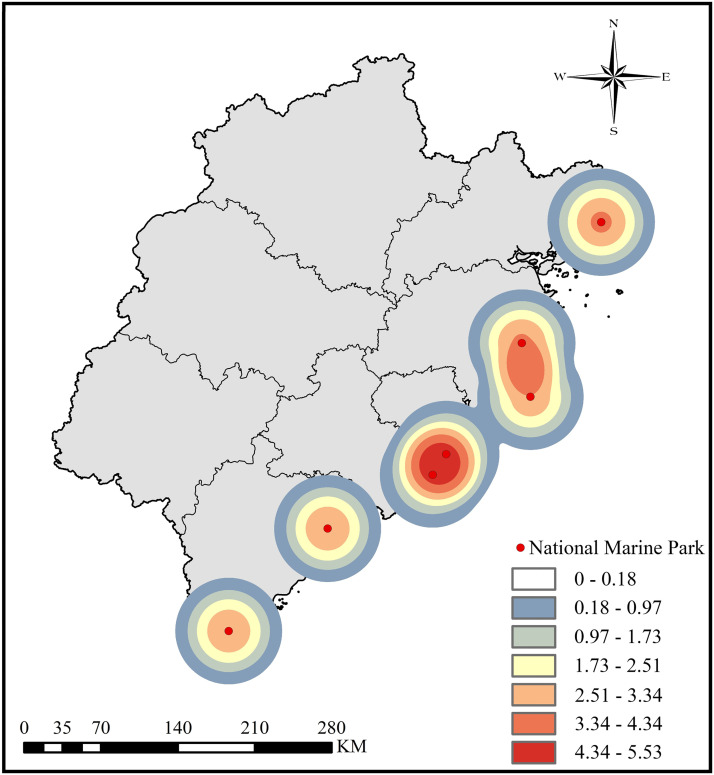
Sentiment Index Distribution.

This study analyzed a dataset consisting of 3,758 images and comments using pre-trained NLP models. The results indicate significant regional differences in sentiment distribution, consistent with earlier findings in academic research [83]. The findings reveal that overall positive sentiments accounted for 87.06%, while the remaining 12.93% were negative. As shown in Fig 3, The sentiment indices derived from NLP analysis were mapped onto geographic coordinates using ArcMap 10.8.1, and kernel density analysis was conducted with the natural break method. The data were divided into seven categories based on value ranges. In the visualization, color intensity transitions from red, representing regions with high sentiment intensity, to blue or white, indicating areas with more dispersed sentiment distribution or lower sentiment indices. Although the landscape characteristics of Fujian’s Coastal National Parks are relatively uniform, the study revealed subtle regional differences in sentiment distribution. Notably, national parks in the central coastal region of Fujian—such as Meizhou Island and Chongwu—exhibit relatively high tourist sentiment indices. Similarly, study sites located near economically developed metropolitan areas also show high sentiment levels; for instance, the sentiment index in Xiamen reaches 8.68. Conversely, except for the Fuyao Islands, Coastal National Parks farther from major cities exhibit lower sentiment indices. For instance, Pingtan Island’s sentiment index is 8.2.

#### 3.1.1. Photographic preferences of tourists in Fujian’s Coastal National Parks.

**Fig 4:**
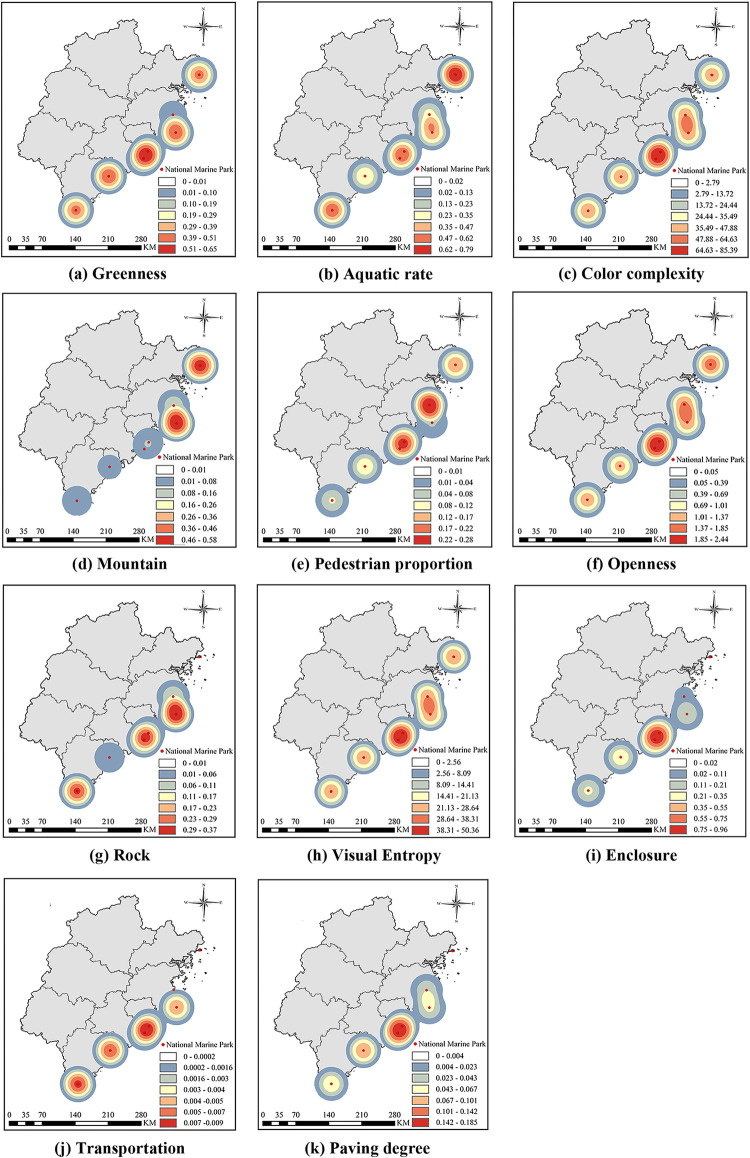
Distribution of Built Environment Data.

Fig 4 illustrates the photographic preferences of tourists within Fujian’s Coastal National Parks based on captured images. Fig 4a indicates the proportion of greenery in images, showing that Coastal National Parks in Xiamen and Chongwu have higher greenness. Tourists tend to prefer images with green landscapes, suggesting that the landscape design and planning in these areas are better compared to others. Fig 4b presents the distribution of aquatic rates, including rivers and seawater. The overall high peak in aquatic rate suggests a potential correlation between aquatic features and positive tourist sentiment. Fig 4c illustrates the complexity of colors within the images, indicating that the national-level coastal parks in Fujian share relatively consistent color elements. As shown in Fig 4d and 4g, mountains and rocks are primarily located in areas with higher landscape complexity. Except for the relatively consistent regions of Pingtan and Changle, noticeable differences in geomorphology exist across Coastal National Parks in other regions. Fig 4e shows the distribution of pedestrian proportions in images, with higher values observed in Changle and Meizhou Island. This indicates poorer transportation accessibility in these areas, leading to increased pedestrian activity. Fig 4f illustrates the openness of the environment in images, with higher values indicating fewer visual obstructions. The openness distribution appears relatively uniform, with Changle and Meizhou Island showing higher values (0.37 and 0.35, respectively). Fig 4h analyzes the visual complexity of different Coastal National Park environments by calculating image entropy. The distribution of visual entropy in Fig 4h closely corresponds to the sentiment indices in Fig 3. Tourist emotions are influenced by the correlation between architecture and landscapes, with high peaks representing areas of maximum landscape complexity. Coastal National Parks exhibit consistent entropy values. Fig 4i displays the enclosure values in images, reflecting whether the architecture in the area is more attractive. The highest values are observed in Chongwu and Meizhou Island (0.15 and 0.11, respectively). Fig 4j shows the transportation distribution in Coastal National Parks. Regions with higher kernel density peaks may face challenges such as inadequate road infrastructure or a lack of parking facilities. Fig 4k presents the proportion of exposed ground in images, including features such as paving, land, and grass. Coastal National Parks with higher peak values provide visitors with more diverse ecological experiences in surrounding natural environments. Notably, in the kernel density maps of features such as walls, rocks, transportation elements, and paving extent, some parks do not exhibit any hotspots. This is primarily because tourists show limited interest in photographing or mentioning these elements on social media, resulting in a low number of related UGC images. Consequently, the corresponding variable values approach zero and are either categorized into the lowest level (white) or fail to form a valid density distribution.

### 3.2. Analysis of spatial feature distribution differences based on SHAP values

**Fig 5:**
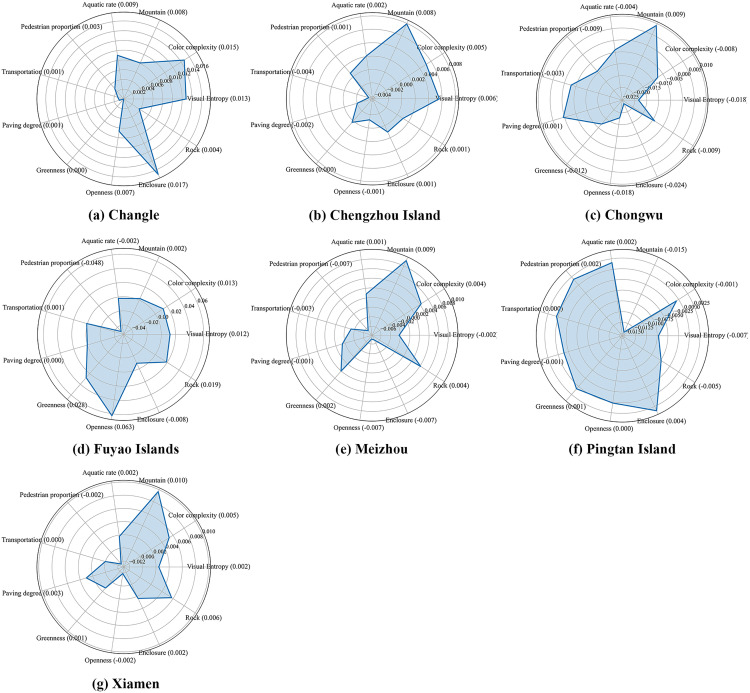
Radar Chart Analysis of SHAP Values.

To further explore the spatial heterogeneity of built environment features in influencing tourist emotions, this study presents SHAP radar charts in Figs 5a through 5g, corresponding to seven national-level coastal parks in Fujian Province: Changle, Chengzhou Island, Chongwu, Fuyao Islands, Meizhou Island, Pingtan Island, and Xiamen. Each chart displays the average SHAP value of 11 model features contributing to sentiment prediction, highlighting the key factors that influence tourist emotions in each park. In Fig 5a for Changle, “enclosure” (SHAP ≈ 0.017) and “color complexity” (≈ 0.015) are the most positively influential features, suggesting that environments with a strong sense of enclosure and rich color information are preferred by tourists in this area. In contrast, features such as “greenery”, “transportation”, and “pavement” have mean SHAP values below 0.002, indicating limited influence on sentiment prediction in this context. In Fig 5b for Chengzhou Island, natural landscape features stand out, with “mountain” (≈ 0.008) and “visual entropy” (≈ 0.006) as primary positive factors. Conversely, “transportation” shows a notable negative value (≈ −0.004), suggesting that infrastructure may have a suppressive effect on tourist emotions, possibly reflecting inconveniences associated with lower levels of urbanization. In Fig 5c for Chongwu, the model reveals a sharp contrast: while “mountain” contributes positively (≈ 0.009), “enclosure” exhibits the most negative value among all features (≈ −0.024), indicating that a sense of confinement significantly affects tourist emotions in this area. Tourists may prefer open and visually transparent environments. The contribution of “pavement” is minimal (≈ 0.001), suggesting it is not a primary explanatory factor for emotional variation. In Fig 5d for the Fuyao Islands, spatial structure perception emerges as the dominant factor. “Openness” has the highest SHAP value across all regions (0.063), followed by “greenery” (≈ 0.028), indicating that wide views and vegetation significantly enhance emotional responses in this area. In contrast, “population density” (≈ −0.048) and “enclosure” (≈ −0.008) show significant negative impacts, suggesting that crowding and spatial compression contribute to negative tourist experiences. In Fig 5e for Meizhou Island, natural features dominate. “Mountain” (≈ 0.009), “rock”, and “color complexity” (≈ 0.004 each) exert consistently positive effects on sentiment. “Openness” and “enclosure” both have negative values (≈ −0.007), suggesting higher tourist sensitivity to spatial boundary characteristics in this region. In Fig 5f for Pingtan Island, the SHAP distribution is relatively balanced, with most feature values ranging between 0 and 0.004. Only “mountain” (≈ −0.015), “visual entropy” (≈ −0.007), and “rock” (≈ −0.005) show significant negative values, indicating that complex terrain and visual overload may disrupt tourist experience and reduce sentiment scores in this area. In Fig 5g for Xiamen, “mountain” (≈ 0.01) and “rock” (≈ 0.006) contribute positively to tourist sentiment, reflecting the appeal of natural elevation differences within the urban landscape. “Population density” and “openness” show slightly negative values (≈ −0.002), suggesting that crowding or limited visual openness may have a minor adverse effect on some tourists.

### 3.3. SHAP feature importance analysis

**Fig 6:**
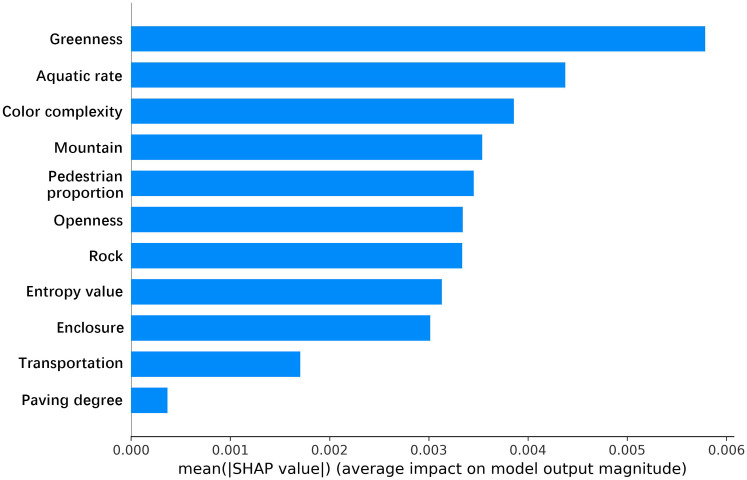
Feature importance in SHAP.

**Fig 7:**
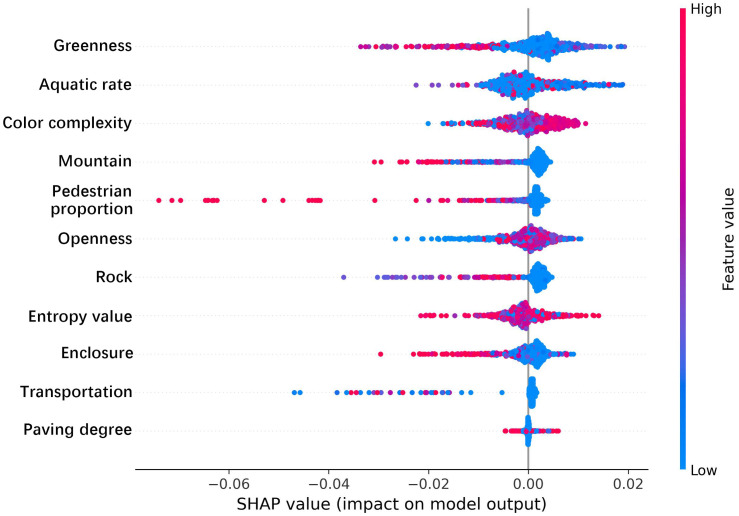
Beesworm distribution of feature importance.

This study employed the LightGBM model and SHAP analysis to investigate the influence of built environment factors and sentiment indices on tourist emotions in national-level Coastal National Parks. Given that the primary objective of this study is to explore the interrelationships and underlying mechanisms among variables rather than to construct a generalized predictive model, the analysis was conducted on the full dataset to maximize data utilization and enhance model interpretability. To improve the robustness and credibility of the results, we additionally evaluated model performance using metrics including the coefficient of determination (R² = 0.9897), root mean square error (RMSE = 0.0300), and mean absolute error (MAE = 0.0143), which indicate excellent model fit. Additionally, the Spearman correlation coefficient (ρ = 0.9011, p < 0.0001) was introduced to further verify the monotonic consistency between predicted values and observed outcomes. The corresponding Spearman R² of 0.8120 suggests a high degree of structural correlation among variables and strong data quality. As shown in Figs 6 and 7, the results of the model analysis were visualized to illustrate the importance and contribution of various research indicators to tourist sentiment indices. In the Fig 6, greenness, aquatic rate, and color complexity are the three most influential variables affecting tourist sentiment indices, with average values exceeding 0.0035. Additionally, six variables—mountain, pedestrian proportion, openness, rock, visual entropy, and enclosure—show relatively lower importance. Finally, the two least significant indicators are transportation and paving degree. Fig 7 presents the corresponding swarm plot, showing the SHAP values for each feature of individual samples. This plot provides a better understanding of overall patterns, where each point represents a sample and the color indicates the feature value (red for high, blue for low). Fig 7 illustrates the bidirectional distribution trends of the greenness feature on model outputs. When feature values are high, the negative impacts are relatively pronounced. In contrast, the distribution of aquatic rate and openness is relatively stable. When feature values are low, negative impacts are observed, whereas higher feature values result in predominantly positive impacts. Notably, the five indicators—mountain, pedestrian proportion, rock, enclosure, and transportation—exhibit similar trends, where higher feature values are more likely to induce negative emotions among tourists. Although color complexity negatively impacts most points in the swarm plot, higher feature values are associated with greater positive impacts on the model. While visual entropy and paving degree generally exhibit high feature values, their impacts on positive and negative extremes are evenly distributed, warranting further analysis. Therefore, in the planning and design of national-level Coastal National Parks, strategies such as rational vegetation design and color coordination, higher proportions of water bodies, open views, avoiding the obstruction of mountains and rocks in route planning, and carefully designed road systems to prevent crowding can effectively enhance tourists’ positive emotions. Overall, Sections 3.1 to 3.3 have provided preliminary analyses of the geographic distribution and feature importance of various research indicators. Nonetheless, while these indicators make significant contributions to the model, their relationships with different sentiment index values remain unclear. To address this, the study will further employ partial dependency plots to explore the interrelationships among these indicators.

### 3.4. SHAP partial dependency plot analysis

**Fig 8:**
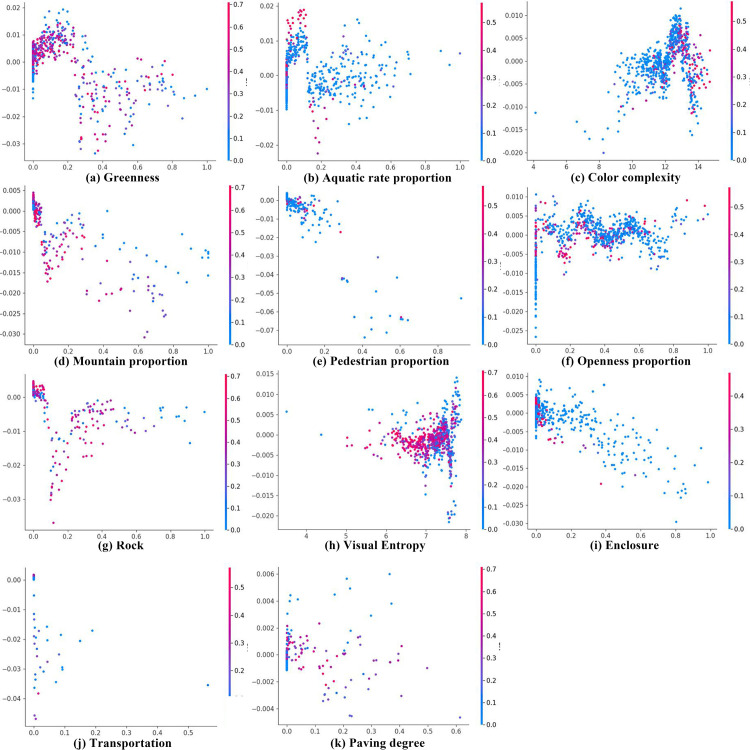
Partial Dependence Plots analysis.

Fig 8 presents the SHAP partial dependency plot analysis, where variables are ranked based on their contribution values in Fig 6. Fig 8a–8c represent the most influential variables in the model. Fig 8a illustrates greenness, where the contribution value reaches a stable peak around 0.2 before showing an overall downward trend. This stability before 0.2 may indicate variations in tourist emotional preferences and positive impacts on their sentiments. When greenness exceeds 0.2, it may indicate the presence of unplanned wilderness landscapes, leading to increased contribution volatility and instability, ultimately resulting in greater negative impacts. Fig 8b indicates that when the aquatic rate is below 0.18, positive contributions are higher, with a data cluster observed. When the aquatic rate exceeds 0.18, a clear upward trend in instability is observed, with contributions predominantly negative. This indirectly suggests a nonlinear relationship between aquatic rate and tourist sentiment indices. Fig 8c depicts color complexity, where positive contribution rates rise sharply between 10 and 13, reaching a peak at 13 before declining rapidly. This marks the critical value at which color complexity impacts tourist emotions in Coastal National Parks. Fig 8d shows the impact of mountain on emotions. When the values are below 0.05, the data are relatively concentrated, with stable positive and negative contributions. However, when values exceed 0.05, contribution values become unstable and highly volatile, predominantly showing negative contributions. This highlights the need to consider the proportion of mountain within the visual range when designing Coastal National Park routes. Fig 8e indicates that pedestrian proportion has a linear relationship with tourist emotions. As pedestrian proportion increases, corresponding negative contributions also grow. Fig 8f illustrates the bidirectional distribution of openness. Contributions remain relatively stable before reaching a value of 0.7, but become unstable once the value exceeds 0.7. Fig 8g describes the role of rock, showing a gradual transition from positive to negative contributions when the values range from 0 to 0.1. Although contributions rise sharply between 0.1 and 0.3, they stabilize beyond 0.3, remaining predominantly negative overall. When planning Coastal National Parks, attention should be given to the proportion of rock as visual elements. Fig 8h shows that most visual entropy values are concentrated in the SHAP negative contribution range, with contributions clustering between 6 and 8. As entropy increases, the distribution of SHAP values becomes more dispersed, indicating that higher entropy may increase uncertainty in the output. Fig 8i reveals a distinct data cluster when enclosure values are below 0.2. However, when values exceed 0.2, the data show an unstable upward trend. This indirectly suggests a nonlinear coupling between spatial scale and sentiment indices. Fig 8j and 8k illustrate less significant indicators. Fig 8j shows that the transportation indicator has an overall negative contribution to tourist sentiments, with significant instability and large fluctuations in its distribution. Fig 8k describes paving degree. When the value is below 0.1, it predominantly has a positive impact on sentiment indices. However, as the value exceeds 0.1, contribution values become more volatile and tend to shift toward negative contributions. This indicates that the planning and construction of Coastal National Parks should preserve sufficient natural surfaces, such as grasslands and beaches, as excessive paving could lead to negative emotional impacts on tourists.

### 3.5. SHAP heatmap analysis

**Fig 9:**
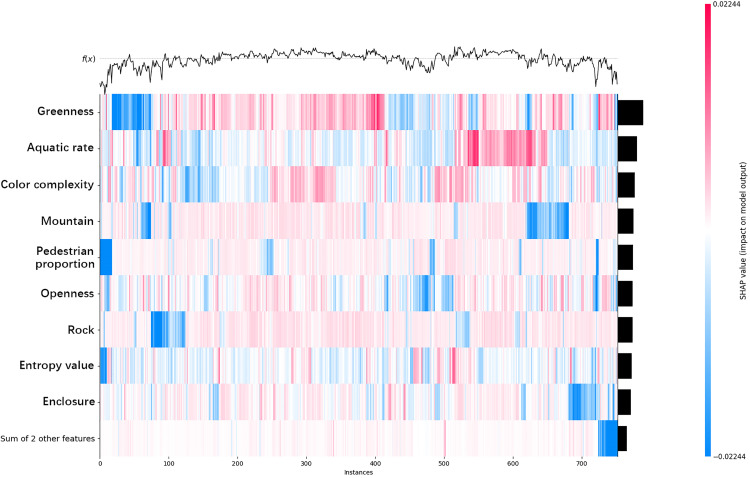
SHAP Heatmap analysis.

Fig 9 presents the SHAP heatmap analysis. Visual elements (e.g., Greenness, Aquatic rate, Color complexity, Mountain, etc.) are listed along the left side of the heatmap, while instances are arranged along the X-axis. SHAP values are color-coded, with red representing positive impacts (i.e., the visual element’s positive contribution to model outputs) and blue representing negative impacts (i.e., its negative contribution). The line graph at the top of the Fig f(x) illustrates the trend of model output values across instances. Combined with the heatmap, it provides a clearer view of how specific features influence emotional perception preferences in certain instances. Firstly, the heatmap shows that SHAP values for Greenness are generally high, with concentrated red areas on the left side, indicating a significant positive impact of vegetation coverage on certain instances. Greenness notably enhances tourists’ emotional perception preferences. The color distribution of Aquatic rate alternates between red and blue, indicating a bidirectional influence on tourists’ emotional perception—it enhances emotional perception in some contexts but may reduce it in others. The SHAP values for Color complexity are relatively dispersed and faint in color, indicating a smaller and more unstable impact on emotional perception. The SHAP values for Mountain show concentrated blue areas, indicating that mountainous features may reduce tourists’ emotional perception in some instances. Pedestrian proportion exhibits significant negative impacts (darker blue areas) in certain instances, suggesting that crowded environments may reduce tourists’ emotional perception preferences. Openness and Enclosure display complex patterns of SHAP values, suggesting that their impacts on tourists’ emotional perception vary depending on the context, without a clear unidirectional preference. The deeper the color, the stronger the influence of the visual element on the emotional perception of that instance. Overall, Greenness and Aquatic rate exert significant influence, while Color complexity and Entropy value (visual entropy) are more dispersed and have weaker impacts. From the Fig, it can be concluded that Greenness and Aquatic rate are the primary visual elements influencing tourists’ emotional perception preferences. The former predominantly exhibits positive impacts, while the latter’s influence varies depending on the context. Mountain and Pedestrian proportion may have negative impacts on emotional perception in certain contexts, while the effects of other visual elements are more complex and lack a clear unidirectional trend. This analysis provides important references for landscape design and urban planning, suggesting that optimizing these key visual elements can effectively enhance tourists’ emotional experiences. Additionally, near the peaks of the f(x) value region, it can be observed which visual elements have SHAP values displayed in red (positive contributions). These elements may be the primary reasons for the improvement in emotional perception preferences. For example, in the Fig 9, when f(x) reaches a peak, Greenness SHAP values may appear prominently in red, indicating that Greenness has a significant positive impact on emotional perception in this instance. Conversely, when the f(x) curve is at a low point, Mountain or Pedestrian proportion SHAP values may appear in blue, indicating that these visual elements negatively impact emotional perception in this instance.

## 4. Discussion

### 4.1. Perspectives on key impact indicators in the built environment of Coastal National Parks

This study conducted a detailed experimental analysis of various visual environmental indicators to determine their effects on tourists’ emotional perceptions. Specifically, greenness, aquatic rate, and color complexity were identified as the most significant environmental indicators influencing tourists’ emotions in Coastal National Parks. While different spatial environments may yield varying results [84], these indicators reveal tourists’ preference tendencies when selecting environmental photos to some extent. Notably, the experimental data indicate that common elements in artificial environments, such as transportation and paving, have relatively weaker impacts on tourists. This trend can partly be attributed to tourists deliberately avoiding such elements when selecting environmental photos, thus limiting their representativeness. This is consistent with previous research, which emphasized the relatively weaker attractiveness of artificial environments [85]. Secondly, the substantial influence of greenness and aquatic rate visibility can be attributed to planning preferences in Coastal National Parks. Tourists’ appreciation of green and aquatic spaces significantly influences planning trends, as appropriate greenness positively impacts health, happiness, and satisfaction levels [86]. However, the unregulated expansion of urban construction indirectly reduces the proportion of green and aquatic spaces, resulting in uneven sentiment distribution in Coastal National Parks. This also helps explain why most Coastal National Parks located on the urban fringe tend to exhibit lower sentiment indices [87]. The study found that the optimal range of greenness for influencing tourists’ emotions is between 0.1 and 0.2. This finding contrasts with the conclusions of previous research by Tomao et al. [88], which suggested that higher greenness levels are more beneficial for human emotions. The fluctuation in the emotional impact of greenness may stem from the interactions among multiple environmental indicators in this study, which make the influence mechanism more complex and unstable. Furthermore, in this experiment, color complexity showed a high contribution rate to the model, further validating the critical role of color in identifying tourist experiences and influencing their emotions [89]. “Comments such as ‘The seawater is breathtakingly beautiful, with a macaron-blue hue, misty waves, and a beach full of seashells—an ideal place for a vacation,’ and ‘Xiamen Island Ring Road has outstanding greenery, with vibrant flowers and lush grass. One side of the road runs along the coast with a beach. This place left me with unforgettable memories!’ from the dataset support this observation.” The experiment revealed that the optimal range of color complexity in the environment of Coastal National Parks is between 12 and 13. However, the experimental results indicate that most photos have a color complexity below the expected range, suggesting that the built environment in Coastal National Parks has room for improvement in terms of color design to more effectively enhance tourists’ emotional experiences.

### 4.2. Recommendations for sustainable development of Fujian’s Coastal National Parks

#### 4.2.1. Infrastructure development in Coastal National Parks.

In experiments conducted in Fujian’s Coastal National Parks, researchers examined the impact of marine natural landscape indicators on emotions. The results revealed that key drivers of positive emotions are often not the richness of vegetation coverage or aquatic rate. Tourists’ experiences are often significantly influenced by specific conditions, such as color complexity, openness, and mountain features. Therefore, incorporating elements of plant diversity in Coastal National Parks, such as applying eco-engineering techniques to restore marine ecosystems through seagrass planting or artificial reef construction, can help restore and enhance marine biodiversity. Monitoring and evaluating the ecological benefits of these measures can provide scientific data to guide long-term conservation plans [90]. Enhancing water richness and increasing color diversity in the environment are crucial for alleviating the visual fatigue caused by monotonous water bodies or greenery, and planners should also pay attention to sky- and building-related indicators because of their positive or neutral effects on tourists’ emotions. Within the tourism areas of Coastal National Parks, strengthening landscape infrastructure, such as minimally invasive floating walkways, eco-floating viewing platforms, and coastal eco-friendly trails, can bring tourists closer to nature without damaging the environment. This strategy is particularly suitable for areas with high ground exposure and openness values identified in the experiments. As pedestrian proportion also significantly affects tourists’ emotions, smart visitor management systems can be developed and implemented using technologies such as thermal imaging, cameras, and Radio Frequency Identification (RFID) to monitor visitor flows in real time. Combined with big data analysis, optimizing visitor route planning can prevent overcrowding and reduce environmental pressure. Finally, dynamic changes in tourists’ perceptions should be fully considered when adjusting tourism attractions in Coastal National Parks. To alleviate pressure on coastal landscapes, measures such as limiting the number of visitors, providing online educational resources, and offering on-site interpretation services can help tourists understand the importance of ecological conservation while reducing negative impacts on the coastal environment [91].

#### 4.2.2. Enhancing visual balance in marine landscapes.

The analysis of experimental results indicates that visual entropy has a relatively balanced impact on tourists’ emotional responses. Additionally, the distribution patterns of most indicators in Coastal National Parks significantly overlap with regions of high visual entropy. Therefore, one of the main challenges in developing Coastal National Parks lies in effectively utilizing areas with high visual complexity, optimizing visual experiences, and thereby enhancing overall tourist satisfaction and eliciting positive emotional responses. However, variations in landforms, vegetation density, and seasonal climate characteristics in the natural landscapes of Coastal National Parks can influence visual complexity, resulting in differences in the landscapes captured in tourists’ photos. Therefore, planning and design should focus on studying the nonlinear relationships between tourists’ emotions and landscape indicators and use big data analysis to determine the optimal threshold ranges. Additionally, planning should prioritize maximizing tourist enjoyment while minimizing environmental impacts. This ensures that tourists can appreciate natural landscapes without harming the marine environment [92]. For example, in ecologically sensitive areas, implementing ecological capacity assessments, introducing environmental education and training signage, or establishing ecological buffer zones can mitigate human disturbances to core areas and ensure maximum protection of marine natural landscapes. Meanwhile, to maintain the originality of rocks and water bodies as well as the stability of plant-related indicators that influence emotions in Coastal National Parks, excessive development should be strictly restricted. This will preserve the positive impact on tourists’ visual perceptions and ensure ecosystem stability, thus sustaining essential ecosystem services [93].

### 4.3. Research contributions

This study quantified textual data and corresponding image data from social media platforms as data sources. It employed LightGBM, SHAP swarm plots, SHAP feature importance analysis, SHAP partial dependency plots, and SHAP heatmap analysis to explore the interactive mechanisms between tourists’ emotions and the built environment’s visual elements in Coastal National Parks. Previous studies in this field typically used urban areas as research sites, relying on 360-degree street view images as data sources [94,95]. Studies focusing on park areas, particularly Coastal National Parks, as research sites are relatively limited. In the context of urbanization, existing model evaluation frameworks need to account for factors such as population density and urban development. However, this study focuses on the built environment in Coastal National Parks, extending the application of sentiment-based tourist evaluation frameworks to built environments. The potential of SHAP to explain machine learning models is critical in the field of quantitative interpretive analysis. Moreover, integrating commonly used SHAP tools, such as dependency plots, feature importance, SHAP heatmaps, and waterfall plots, provides a more comprehensive understanding of how individual variables influence the overall model [96]. Specifically, analyzing SHAP swarm plots, such as those in Fig 6, reveals each feature’s contribution to prediction results, intuitively displaying their importance and the distribution of SHAP values under different prediction values. These visualizations help researchers understand how the model makes predictions based on different features while revealing the interactions among features, thereby enhancing the model’s interpretability. This highlights the advantages of machine learning in complex data analysis, introducing a more advanced framework for element analysis that is critical for guiding the planning and design of Coastal National Parks, as well as their daily maintenance and inspection. Additionally, the GIS technology applied in this study is also noteworthy. While previous studies systematically evaluated urban landscapes using GIS technology [97]. However, this study integrates kernel density estimation with these maps, combining map visualization with machine learning techniques. This innovative research approach is particularly suitable for assessing the value of small but widely distributed research areas like Coastal National Parks. This study proposes innovative solutions and analytical strategies aimed at promoting sustainable development in the environments of Coastal National Parks.

### 4.4. Research limitations and future directions

This study utilized machine learning techniques and UGC from social media platforms to investigate the relationship between the environment of Coastal National Parks and tourists’ emotions, uncovering important insights. However, this study also acknowledges certain limitations. Although UGC on modern social media platforms has become a reliable and comprehensive resource for decision-making [98]. However, policy restrictions and data security concerns may hinder data collection, affecting the analysis of emotional differences among tourists of different genders, age groups, or geographic regions [99]. This limitation may fail to fully capture the preferences of Coastal National Park visitors or other groups, restricting the generalizability of the findings and leading to a bias toward tourists who frequently use tourism social media platforms. Therefore, to address these limitations, research projects should incorporate diverse information sources such as online surveys and demographic classifications.

The method of extracting sentiment data from online platform photos demonstrated high prediction accuracy in machine learning models. However, the results may still exhibit localized biases, as human emotional perception is influenced not only by visual factors but also by a wide range of sensory inputs, including taste, smell, hearing, and touch [100]. Additionally, datasets constructed from user feedback or image content may be insufficient to accurately reflect and represent human emotional states, potentially introducing biases when analyzing tourist emotions. Therefore, to enhance the rigor of research findings, future studies should incorporate field surveys to ensure coverage of a broader range of tourist groups, beyond active online users. Furthermore, future research may need to integrate methods such as offline interviews, public surveys, and machine learning results to more comprehensively and accurately evaluate and understand the diversity of tourists’ emotional states.

During the experiment, the collection and processing of social media image data overlooked dynamic factors affecting tourists’ emotions, such as daily variations, different weather conditions, seasonal factors, and subjective or objective influences during photo-taking. The experiment employed a binary classification method to categorize emotions into positive and negative, which simplified the complexity of emotional experiences and failed to capture more nuanced emotional states such as joy, awe, satisfaction, and excitement [101]. Future research should consider using more detailed emotion classification datasets to train more accurate emotion prediction models. Although this simplified approach makes the findings easier to compare with existing literature, it limits the ability to identify and predict subtle emotional responses in tourism. Therefore, capturing neutral emotional response trends that may arise during tourists’ experiences in Coastal National Parks becomes particularly challenging. To comprehensively study the diversity of tourists’ emotions and their underlying mechanisms, future research can employ multi-class datasets.

Finally, this study explored how the landscape environment of Coastal National Parks influences tourists’ perceived emotions and visualized the experimental data using ArcMap software. Due to the influence of complex environmental factors, tourists may perceive the same image differently. While this experiment visualized the regional distribution of tourists’ emotions using ArcMap’s kernel density analysis, it may not fully represent the diverse emotional perceptions of each individual tourist. Therefore, future research could employ more detailed methods to analyze geographic information visualization and incorporate analyses of temporal and spatial changes in tourists’ emotional perceptions. This will further deepen our understanding of how Coastal National Parks influence human emotions and provide guidance on leveraging this knowledge to promote sustainable marine tourism practices, ultimately benefiting both tourists and the natural environment.

## 5. Conclusion

This study, using Fujian’s Coastal National Parks as a case study, proposed a reliable sentiment evaluation framework for guiding landscape planning in Coastal National Parks. We collected and cleaned textual and image data from tourism social media platforms, utilized NLP models based on the ERNIE-pretrained BERT and RoBERTa architectures, and constructed a regression machine learning model using the LightGBM algorithm to establish an interaction model between tourists’ emotions and environmental indicators. Additionally, the research results were visualized using ArcMap software. This study identified key factors in the landscape environment of Coastal National Parks that influence tourists’ emotions and proposed strategies for the sustainable optimization of marine-themed landscapes. The main findings are as follows:

Analyzing textual and image data collected from tourism social media platforms revealed that 87.06% of overall emotions were positive, while 12.93% were negative. (1) The highest average sentiment indices were observed in the Fuyao Islands, Changle, and Xiamen, at 0.92, 0.91, and 0.86, respectively. Conversely, the four lowest sentiment indices were observed in Chengzhou Island, Meizhou Island, Pingtan Island, and Chongwu, at 0.85, 0.83, 0.82, and 0.74, respectively. This distribution highlights the spatial heterogeneity of emotions across Fujian’s Coastal National Parks. (2) According to SHAP analysis, the most critical ecological indicators, in order of importance, are greenness, aquatic rate, color complexity, mountain features, pedestrian proportion, openness, rock, visual entropy, enclosure, transportation proportion, and paving degree. In planning and designing Coastal National Parks, the optimal range for greenness should be between 0.1 and 0.2. Additionally, aquatic rate should range from 0 to 0.1 and 0.3 to 0.8, color complexity should be between 12 and 13, and mountain proportion should be less than 0.15. Meanwhile, pedestrian proportion, openness, and visual entropy should not be overlooked, as they may have unstable impacts on different individuals. (3) The experimental results highlight the importance of considering not only the individual effects of environmental elements on tourists’ emotions but also the interactive and nonlinear impacts of different environmental factors in the planning of Coastal National Parks. Positive emotions dominated in this experiment, which is a highly encouraging indicator of sustainable tourism development in marine areas. To maintain this advantage and further enhance tourists’ perception of future marine ecosystems, we recommend prioritizing the coordination of visual elements and the layout of infrastructure in the development of Coastal National Park tourism areas, thereby maximizing the unique appeal of marine landscapes. In practical planning and management, attention should be given to the potential for overly homogeneous green vegetation to cause visual fatigue, which could further impact tourists’ emotions.

In conclusion, developing interdisciplinary and adaptive conservation strategies, preserving original built environments, optimizing visual design, planning infrastructure, and effectively utilizing rural landscape resources are key prerequisites for ensuring the sustainable development of marine natural landscape systems. This study emphasizes the importance of precise built environment indicators on emotional responses in Coastal National Parks, addressing gaps in prior research on landscape visual environment indicators. The findings of this study provide practical recommendations for the evaluation and conservation of international Coastal National Park landscapes, with the potential to positively and profoundly impact the sustainable development of global marine ecosystems.
